# The repeated emergence of asexuality, the hidden genomes and the role of parthenogenetic rare males in the brine shrimp *Artemia*

**DOI:** 10.1186/s40709-018-0078-2

**Published:** 2018-05-18

**Authors:** Theodore J. Abatzopoulos

**Affiliations:** Department of Genetics, Development & Molecular Biology, School of Biology, Aristotle University of Thessaloniki, 54124 Thessaloniki, Greece

**Keywords:** *Artemia*, Rare males, Asexual, Evolution, Vectors of haploid genomes

## Abstract

The backbone of this endeavour consists of three major components as they appear in the title. My intention is to summarise, as explicitly as possible, both existing and novel data on the occurrence of parthenogenetic rare males assessing their role in conveying sets of genetic information between asexual strains and sexual *Artemia* species to and fro. Additionally, an assemblage of strong indications and evidence is quoted aiming to unravel possible scenarios of the repeated emergence of asexuality in the brine shrimp and its significance in evolutionary processes involved in speciation.

## Introductory note

The repeated emergence of asexuality has been a long-lasting quest for biologists. The mechanisms of its causes are still debated or erroneously addressed. The controversy originates from our inability to attribute safely whether this issue is the outcome of genetic accidents or the result of established procedures. Yet, nowadays, there are new approaches, offering more persuasive explanations than in the recent past. The new sets of tools are mainly based on molecular markers which may track down either changes both in coding and non coding regions or reveal the existence of entire haploid genomes; I call these genomes, being hosted in hybrids, ‘hidden genomes’. To convey these long sets of information one needs to find the appropriate vehicle for this transport. There is strong evidence that the mediators for such an action are, among few others, the males appearing rarely and sporadically or erratically in obligate asexual populations, most probably due to upsets in meiotic or mitotic procedures, which for all these reasons I shall call, from now on, ‘Parthenogenetic Rare Males’ or PRMs. In this review, I shall focus on disclosing the often-misinterpreted role of these males as transport agents of long genomic regions resulting finally in the recurring emergence of asexuality. I shall base my essay on *Artemia* since the brine shrimp is considered as a model organism for such scientific ventures and for many reasons that are described below in detail.

The genus *Artemia* comprises six sexual species and a complex of asexual populations [1, 2] or agamospecies (for definition see [3]). As feral populations, two of the sexuals are found in the New World (i.e. *A. franciscana* and *A. persimilis*) and the rest (i.e. *A. salina*, *A. urmiana*, *A. tibetiana* and *A. sinica*) inhabit Old World saline-hypersaline water bodies while parthenogenetic populations occur exclusively in the Old World (for more information on *Artemia* biogeography see [4, 5] and references therein). Thus, *Artemia* distribution is expansive, i.e. coastal and inland saline catchments scattered in all continents except Antarctica. One of the most remarkable features of *Artemia*, serving ideally the purpose of this effort, is that there are no species or populations which may switch from one mode of reproduction to the other (from sexuality to parthenogenesis and vice versa); in this way, many interfering ambiguities are eradicated. Another important characteristic of asexual *Artemia* is the existence of different ploidy levels which are undisputedly related to automixis, ascribed mainly, if not exclusively, to diploid parthenogens and apomixis, attributed to triploids and tetraploids (for extensive reviews see [6–8]). It is notable that the absence of ‘innate mate recognition’ (for term definition see [9]) even between distantly related *Artemia* species [1, 10] accommodates hybridization and facilitates gene flow (introgression) among highly differentiated entities in the genus. Besides, *Artemia*, and especially asexual populations, are prone to mishaps during cell division (mitosis or meiosis) which result in chromosomal aberrations (aneuploidies or heteroploidies) tolerably compatible with survivorship of the bearer [7, 11]; this inclination will be discussed in the light of the emergence of rare males in parthenogenetic populations. Finally, taking into account that encysted *Artemia* embryos or newly born nauplii may generate rapidly propagating and easily cultured experimental/laboratory populations or clonal lineages initiated by a single parthenogenetic female, the set of advantages, which render this hypersaline anostracan paradigmatic for this kind of studies, becomes manifest.

As a prelude of what follows I would like to annotate the three major topics which will be unraveled in the next issues: (i) the milestones of rare male occurrence in parthenogens focusing on *Artemia* asexuals, (ii) their specific role as vectors of genetic information, and (iii) a model for repeated emergence of asexuality based on the theory of ‘hidden genomes’ inherent in hybrids. It would be frivolously incorrect if this effort was meant to initiate/address the contribution of contagious parthenogenesis, a fact successfully anticipated by other scientists [12]; the scope of this effort is to present a potential scenario, happening in nature, for the repeated emergence of parthenogenesis in wild, coexisting *Artemia* populations, which may be broadly based on contagious parthenogenesis but not only. The hypothesis is extended to the following: these haploid genomes may be transmitted to functional sexual females (called here hybrids) that may convey them to next generations and when mate with PRMs give new parthenogenetic lineages.

## The role of parthenogenetic rare males as vectors of genetic information

In the weevil *Otiorrhynchus scaber* or other species (i.e. the isopod *Trichoniscus elisabethae*, obligate sexual or obligate asexual), PRMs are produced in low numbers; crosses between diploid or triploid asexual females and RPMs give rise to triploid and tetraploid offspring, respectively [13].

Considering *Artemia*, von Siebold [14] referred for the first time on *Artemia* rare males. Artom in [15] reported the incidence of PRMs in a parthenogenetic *Artemia* population from Cagliari (Italy) with frequency 4/100.

Several investigators seem to avoid capitalizing on readership eagerness to learn about the potential role of rare males. Or in other words, very few dared to touch or deal with this really fascinating but also thorny issue; among those who pioneered the studies on the role of PRMs in *Artemia* is the team of Cai in China (see [16] and references therein). The publications of Bowen and Browne are, also, among the very few addressing this ‘mystic’ item [17, 18]. I would not be fair enough if I concealed the numerous informal discussions I had with many *Artemia* experts during international meetings or symposia, over the last 30 years, on this tenacious quest.

The need for production of PRMs remains contentious since their role is not clearly accommodated in evolutionary processes. Nearly all of the existing literature (related to PRMs, ploidy levels and apomixis/automixis) ends up with the conclusion that ‘the role of PRMs remains obscure’ [19–24] although they are broadly considered functionally fertile. A reliable approach for addressing this important key-factor is to try to formulate a persuasive hypothesis related to the adaptive advantages, which arise for both asexual forms and sexual species from the exchange of numerous sets of genes. It may stating the obvious for asexuals since they are thought of as seriously deprived from effective differentiation mechanisms compared to their sexual relatives. Yet, it is not that pronounced for sexual species unless they become better colonizers in the extreme and oscillating environments they are challenged to settle. The parthenogenetic component, hosted in hybrids as hidden haploid genome, seems to serve adequately this purpose; Bowen et al. [17], based on haemoglobins, presented persuasive evidence (see also below).

Is there solid evidence for gene transfer by PRMs? The first reliable citation on the role of PRMs comes in one of Bowen’s seminal works on *Artemia* genetics [17]. She and her collaborators used haemoglobins as markers to demonstrate that a parthenogenetic rare male from Yamaguchi population (Japan) succeeded in transferring genes to a zygogenetic female belonging to *A. urmiana* [17]. It is noteworthy that this team was extremely careful to generalise this fact to other genes and especially those related to parthenogenetic reproduction. They also delineated that asexuality is not necessarily associated with uniformity (see also [13]). The exclusion of pseudogamy (gynogenesis) is a *sine qua non* of avoiding extensive confusion (for special cases see [25] and references therein and for *Artemia* see [17]).

McDonald and Browne [26] proposed three possible explanations for the production of males by *Artemia* parthenogenetic females: (i) they are functionally sterile and signify a ‘disadvantageous trait’ which will attract a negative selection, (ii) they are sterile and non-functional within their own population and, therefore, their production is related to a genetic trait which is itself adaptive, and (iii) the production of these males is ‘directly advantageous to the parental female’. I will attempt to annotate these three options trying to disclose their potential evolutionary impact.

Nowadays, the majority of the existing literature supports that PRMs are functionally fertile; therefore, they are not considered as reproductive mishaps. This idea was the outcome of ‘unfortunate’ or inappropriate crossings (i.e. *A. franciscana* x parthenogenetic males). There are, at least, two crucial parameters/items to call attention to: (i) parthenogenetic *Artemia* is proven to be phylogenetically related to certain bisexual *Artemia* species, e.g. *A. urmiana, A. sinica* and *A. tibetiana* (see [2, 8, 27] and references therein); thus, the females used in such crossings must belong to species which share common ancestors with parthenogenetic strains producing PRMs, and (ii) the PRMs used in such crossings must be upon their early reproductive maturation since if they do not copulate for several days they become sterile, which is a common fact for all *Artemia* males. Scrutinising these features, the production of PRMs seems to be directly advantageous to the parental female provided that some prerequisites are met: the population they appear is a mixed one and comprises “suitable” bisexual species and asexual strains (see above), the spatial distribution and the reproductive period of both population components (bisexual and asexual individuals/strains) coincide, and PRMs reproductive rigour is equally close to that of bisexual males. Thus, they may contribute in some way (either genetically, or nongenetically, e.g., via gynogenesis or by increasing brood size), to the proliferation or survival of the enriched genome. This is an effective way of transmitting advantageous characters from parthenogenetic to bisexual females provided that all prerequisites are met.

## The recurring emergence of asexuality (the theory of hidden genomes)

The fairly extensive sampling in [27], which aimed to conceive the first global phylogeny of the genus *Artemia*, revealed the existence of at least four independent origins of parthenogenesis with the most ancient dating back to ~ 3.5 MYA. This recurring emergence of asexuality is certainly an underestimate of the reality for many obvious reasons. However, the advantages of using *Artemia* as a model organism for exploring the interactions between sexuality and asexuality are highlighted. The established affinity of parthenogenetic *Artemia* with Asian bisexual species (i.e. *A. sinica*, *A. urmiana* and *A. tibetiana*) implies that PRMs have significant probabilities for being cross-fertile with these species [1, 2, 16, 17, 27]. Moreover, there are many parthenogenetic strains in proximity with the Asian bisexual *Artemia* species forming mixed populations (for example, see [28] and references therein). In this sense, hidden haploid genomes may be expressed and emerge from time to time when genes responsible for the determination of asexuality come to homozygosity. This phenomenon, although rare, is not at all improbable. Considering the above facts, the cradle of recurring emergence of parthenogenesis must be placed in central and southeast Asia. Also, it is crucial to mention that there are three mechanisms present in *Artemia* that may distort the molecular clock and create confusion about polyphyly or monophyly in agamospecies and these are: large population sizes, polyploidy and genome protection by the prolonged dormancy of the encysted embryos ([2] and references therein).

## The validation of this hypothesis

The testing of a hypothesis is imperative to consolidate its validity. Therefore, referring to the present hypothesis, there are certain prerequisites to consider for judging its testability: (i) obligate parthenogenesis is a fact in *Artemia* genus [6], (ii) automixis in diploid parthenogenetic strains and apomixis in polyploid asexual *Artemia* have been confirmed long ago [6, 7], (iii) cytogenetic studies and methodologies summoned to determine ploidy levels in *Artemia* have been reported many times in the past ([11] and references therein), (iv) *Artemia* is compatible with aneuploidy while genome porosity loosens species integrity [10, 11], (v) multidisciplinary approaches (based on morphology and genetic markers) for finger-printing *Artemia* populations have been deployed and extensively used [29], (vi) laboratory cultures initiated even by a single female are routinely maintained and easily monitored [30], (vii) cross-breeding experiments can be easily performed and are advisable to explore the genetic introgression between different *Artemia* entities [2, 17], and (viii) the existence of mixed populations have been documented in many cases around the globe [28, 31, 32]. All the above facts, although sometimes are laborious, may promote the verification of this hypothesis if properly and collectively addressed.

## Concluding remarks

This is the first time that a potential scenario about the role of PRMs in the evolutionary make-up of *Artemia* genus is attempted. I have focused on presenting the environmental and population components and factors that may facilitate the evolutionary potential and adaptive significance of PRMs in the genus *Artemia* if and when they appear. Their role is envisaged as possible vehicles of haploid genomes, which may either enhance the existing adaptedness or promote recurring emergence of asexuality. Such an effort would definitely involve extensive sampling and biomonitoring and would be very laborious and demanding but by no means unfeasible.

We have to be very cautious when attributing the production of PRMs to certain females, which bear this unique characteristic and transmit it to the new lineage/s (sexual or asexual); only in this case, we would be entitled to refer to a “trait”. I tend to consider this outcome as a mishap, which, however, may have an evolutionary impact to both sexual and asexual bearers rendering the former better colonizers and giving to the latter the opportunity of recurrence. This controversy may sound as an oxymoron. After all, evolution is largely based on ‘accidents’ that if are advantageous they may become established.

Finally, I consider central and southeast Asia as the cradle of the emergence of *Artemia* parthenogens. Topologically and chronologically, this area appoints many of the required features to support this phenomenon; the utmost one is that it hosts parthenogenetic strains together with their phylogenetically close sexual relatives as mixed populations. It is neither circumstantial nor accidental that asexual *Artemia* has never been documented as natural population in the New World.
